# Performance of natural product-based materials as adhesives in the fabrication of mangrove wood composites^☆^

**DOI:** 10.1016/j.heliyon.2023.e13032

**Published:** 2023-01-18

**Authors:** Damilola Oluwafemi Samson, Ahmad Shukri, Nurul Ab. Aziz Hashikin, Siti Hajar Zuber, Abdul Dahiru Addo Buba, Mohd Zahri Abdul Aziz, Rokiah Hashim, Mohd Fahmi Mohd Yusof, Sylvester Jande Gemanam, Peter Ayoola Samson

**Affiliations:** a School of Physics, Universiti Sains Malaysia, 11800, USM, Malaysia; b Department of Physics, University of Abuja, 900211, Abuja, Nigeria; c Advanced Medical and Dental Institute, Universiti Sains Malaysia, 13200, Bertam, Malaysia; d School of Industrial Technology, Universiti Sains Malaysia, 11800, USM, Penang, Malaysia; e School of Health Sciences, Universiti Sains Malaysia, 16150, Kota Bharu, Kelantan, Malaysia; f Department of Physics, Benue State University, 102119, Makurdi, Nigeria; g Department of Chemistry, University of Abuja, 900211, Abuja, Nigeria

**Keywords:** Soy protein-based products, Adhesive, Biomaterial, Composite, Physiochemical characteristics

## Abstract

Biodegradable adhesives prepared using three different forms of soy protein-based products (defatted soy flour/soy protein concentrate/soy protein isolate), sodium hydroxide, and itaconic acid polyamidoamine-epichlorohydrin (IA-PAE) with 0 wt%–20 wt% substitution rates were utilized to enhance the production of mangrove wood composites. ^1^H nuclear magnetic resonance, differential scanning calorimetry, and ultra-high-resolution field emission scanning electron microscopy were employed to characterize the composite samples. Other measurements involved the determination of viscosity, pH, physical, mechanical, dimensional stability, CT numbers, and relative electron density parameters. The ideal curing conditions for the composite bio-adhesives were found to be 15 wt% IA-PAE, 602.50 ± 172.21–391.11 ± 105.82 mPa s, pH 11.0, 180 °C, and 18 min, respectively. The improved physiochemical characteristics of DSF, SPC, and SPI confirmed that NaOH/IA-PAE was integrated into the adhesive system and ameliorated the overall performance of the resulting composites. The results showed that all composite samples, except for those bonded with 0 wt% and 5 wt% IA-PAE, matched up with the quality specification stated in the JIS A-5908 and ASTM D1037. Samples D1, D2, and D3 exhibited optimum characteristics, demonstrating their uses in the development of low-toxicity and sustainable reference tissue substitute phantom in radiological areas.

## 1 Introduction

There has recently been a lot of research in the radiation dosimetric phantom literature regarding the suitability of water or paraffin wax as reference tissue substitute materials. It is generally accepted that these tissue-mimicking materials must be chemically and physically stable over time and with temperature changes, as well as being readily available and adaptable to give contrast. However, paraffin waxes deviate from these tissue equivalence at low energies, they interfere with covalent interfibre bonding by radicals and other reactive functional groups on fibre surfaces [1]. It also reduces the formation of hydrogen and other non-covalent bonds. As a result, the mechanical properties of wood composites containing paraffin waxes were inferior to those made without paraffin waxes [2]. On the other hand, because of the difficulties in regulating the temperature and humidity of the water phantom and its surroundings, as well as the water container's vulnerability to mechanical instability, the use of liquid water can be daunting and inconvenient in some cases. Solid homogeneous phantoms made of biological materials which can be fabricated into composite particleboards have been demonstrated to have unprecedented dosimetric characteristics of water [3–5]. But the limited temporal stability and easy degradation of these phantom materials frequently result in a restriction in the specificity of heterogeneities or intricate layered structures of the biological tissues to be mimicked [6]. In nature, wood and wood products are good examples of a natural composite in which cellulose fibres are glued together in the matrix, reducing composite toughness [7]. Mangrove *Rhizophora* spp. wood, it is a highly attractive material for use as an active tissue substitute in several medical health applications [4,5,8]. However, without modifiers, the amounts of *Rhizophora* spp. particleboards are ineffective, so the use of suitable adhesive materials with unique properties is recommended [[3], [4], [5],^7^,^8^].

Petroleum-derived formaldehyde-based adhesives are still the most widely used adhesives for various types of wood-based composites, especially in particleboard and medium density fibreboards [9]. However, stringent environmental and human health safety regulations, as well as increasing raw material costs, have prompted researchers to minimize the amount of hazardous substances and/or costly adhesive components in finished products and to replace conventional adhesives with more environmentally friendly and safer alternatives [7,10,11]. The use of formaldehyde-free bio-based adhesives, which do not substantially raise manufacturing costs while resulting in wood-based composites with suitable properties, is one of the most innovative “green” solutions to address this issue [12–15]. Renewable resources such as soy protein-based products are essential in the composite particleboard industry, however, there has not been much advancement or new innovation on this research topic relating to the tissue substitute phantom in radiological regions concerned in the existing documented literature [5,12,14,[16], [17], [18]].

Soy protein-based adhesives, as renewable resources, include three basic types: defatted soy flour (DSF), soy protein concentrate (SPC), and soy protein isolate (SPI), and are made up of albumins and globulins with four distinct main water-extractable fractions: 2S, 7S (β-conglycinin, γ-conglycinin, and basic 7S globulin), 11S (glycinin), and 15S (letter S denote Svedberg units), as well as constitutes 35–80 wt% total protein content [9,19]. Despite several attempts to use untreated DSF, SPC, and SPI successfully, the major obstacle is the inappropriate cohesive parameters, particularly poor water-resistance and bonding strength, high viscosity, low solid content, inhomogeneous nature, and inability to cure quickly enough to allow for structural stability [20,21]. Furthermore, these high-viscosity adhesives have limited technical applicability, such as difficulty brushing onto wood composites, low wettability, and a long hot-press period, necessitating the use of at least one curing agent to achieve the characteristics of the materials needed for tissue equivalence [22]. Strengthened materials via modification techniques have promised to enhance soy protein-based adhesives [23]. However, there is a lack of experimental evidence to understand the effects of modification techniques in the wood composite industry; chemical cross-linking is by far the most common approach. A recent study explores the various laboratory procedures based on the treatment of sodium hydroxide (NaOH)/itaconic acid polyamidoamine-epichlorohydrin (IA-PAE) with DSF/SPC/SPI, which results in high strength with low viscosity, non-toxicity, and high water-resistance, characteristics that prevent delamination and provide excellent temperature stability [5,[12], [13], [14],^16^,^17^,^21^].

Thus, the present research is concerned with investigating the adhesive physiochemical characteristics, physical, mechanical, and dimensional stability properties, as well as the effects of temperature and time on different formulations of DSF, SPC, and SPI-bonded *Rhizophora* spp. wood composites based on NaOH (10 wt%) and IA-PAE substitution rates ranging from 0 wt%–20 wt% of varying pH.

## 2 Materials and methods

### 2.1 Adhesive preparation and development of particleboards

DSF, SPC, and SPI (200-mesh, moisture content of 5–5.7%, and ≤ 74 μm of particle sizes) were procured from Wachsen Industry Company Limited in Qingdao, China. The Forestry Department of the Mangrove Forest Reserve, Kuala Sepetang in Perak, Malaysia, generously provided raw *Rhizophora* spp. tree trunks (moisture content of 5–8%). Sodium hydroxide was obtained from Sigma-Aldrich Co., Ltd (USA). IA-PAE adhesive was synthesized using a chemical cross-linking technique as utilized in a previously reported study by Samson et al. [5], which verified its validity as a low viscosity aqueous solution that could be miscible with DSF, SPC, and SPI mixtures to form a stable and solution-like adhesive.

The raw *Rhizophora* spp. tree trunks were grounded and separated into particle sizes (≤ 74 μm) following the method by Samson et al. [5]. The selected particle size was chosen because larger particles exhibit a higher moisture content, while smaller particles contain lower amounts of moisture. All samples were formulated by dissolving the necessary amount of DSF-, SPC-, and SPI-based adhesives in distilled water for 30 min at 25 °C while being vigorously stirred at 600 rpm. Five different IA-PAE substitution rates were used to find the best adhesive formulation for *Rhizophora* spp. composite development. IA-PAE (0, 5, 10, 15, and 20 wt%) and 2 N NaOH (10 wt%, pH of 11.0) were carefully applied to the slurry mixture and stirred at room temperature for 30 min until a color shift was detected, after which they were ready to be used in composite processing. Following the representative procedure for the composition of the total quantity of DSF-, SPC-, and SPI-NaOH/IA-PAE adhesives and distilled water as coded in Table 1, fifteen different adhesive samples were prepared.

**Table 1:** Experimental design for different adhesive mixture.

Adhesive code	Sample description	Approximate adhesive content (wt%)
A1	Uncured DSF	DSF (25); distilled water (75)
A2	Uncured SPC	SPC (20); distilled water (80)
A3	Uncured SPI	SPI (12); distilled water (88)
B1	DSF/NaOH/IA-PAE (5)	DSF (25); distilled water (60); NaOH (10); IA-PAE (5)
B2	SPC/NaOH/IA-PAE (5)	SPC (20); distilled water (65); NaOH (10); IA-PAE (5)
B3	SPI/NaOH/IA-PAE (5)	SPI (12); distilled water (73); NaOH (10); IA-PAE (5)
C1	DSF/NaOH/IA-PAE (10)	DSF (25); distilled water (55); NaOH (10); IA-PAE (10)
C2	SPC/NaOH/IA-PAE (10)	SPC (20); distilled water (60); NaOH (10); IA-PAE (10)
C3	SPI/NaOH/IA-PAE (10)	SPI (12); distilled water (68); NaOH (10); IA-PAE (10)
D1	DSF/NaOH/IA-PAE (15)	DSF (25); distilled water (50); NaOH (10); IA-PAE (15)
D2	SPC/NaOH/IA-PAE (15)	SPC (20); distilled water (55); NaOH (10); IA-PAE (15)
D3	SPI/NaOH/IA-PAE (15)	SPI (12); distilled water (63); NaOH (10); IA-PAE (15)
E1	DSF/NaOH/IA-PAE (20)	DSF (25); distilled water (45); NaOH (10); IA-PAE (20)
E2	SPC/NaOH/IA-PAE (20)	SPC (20); distilled water (50); NaOH (10); IA-PAE (20)
E3	SPI/NaOH/IA-PAE (20)	SPI (12); distilled water (58); NaOH (10); IA-PAE (20)

Remark: DSF = defatted soy flour, SPC = soy protein concentrate, SPI = soy protein isolate, NaOH = sodium hydroxide, IA-PAE = itaconic acid polyamidoamine-epichlorohydrin; 0, 5, 10, 15, and 20 wt% indicate IA-PAE substitution rate; 12, 20, and 25 wt% refer to SPI, SPC, and DSF content; 10 wt% denote NaOH content; 45, 50, 55, 58, 60, 63, 65, 68, 73, 75, 80, and 88 wt% depict distilled water content.

Each sample formulation was mixed for 10 min in a high-speed mixer at a rotating speed of 25000 rpm, then placed into a mould (30 × 30 cm^2^) and pre-pressed for 5 min at 0.49 MPa and 25 °C. After that, a laboratory hydraulic press was used to hot-press the material to a thickness of 1.0 cm and a density of 1.0 g/cm^3^ for 18 min at 20 MPa and 180 °C. When the effects of hot-pressed time and temperature on composite sample parameters were studied, they were varied to ensure optimization. Five replicates were used for each adhesive-bonded composite sample, totaling 75, and the quality specifications stated in JIS A-5908:2015 and ASTM D1037:99 were applied as reference values [24,25].

### 2.2 Effect of viscosity and solution pH

A DV-II + Pro viscometer (Brookfield, Middleboro, MA, USA) was used to measure the apparent viscosity of the adhesive slurries under investigation at 100 rpm, 25 °C, and 55% RH. The viscous drag of the fluid against the spindle was ascertained by the deflection of the spring, which was then evaluated by a rotary transducer. Approximately 0.5–2.0 mg of each adhesive sample was prepared and placed in the sample cup, which was then connected to the viscometer. Likewise, pH values for the cured adhesive samples were determined using a pH meter (Sartorius PB-10 Standard), calibrated via standard buffer solutions of pH (4.01 ± 0.01, 7.00 ± 0.01, and 10.00 ± 0.01) at 25 °C–27.58 °C range in order to extend the duration of the tests [26]. The reported values of apparent viscosity and pH are the mean of five replications.

### 2.3 Physiochemical characteristics

#### 2.3.1 ^1^H NMR test

To elucidate the IA-PAE resin structure, ^1^H NMR measurements were carried out and permitted signals assignment of IA-PAE structure. Liquid state ^1^H NMR spectra were obtained using a Bruker AV-III 500 spectrometer (BioSpin AG, Fällanden, Switzerland) fitted with a 0.5 cm cryoprobe at 500 MHz and 25 °C. Chemical changes were accomplished by dissolving adhesive solutions (0.01 g) in small quantities of heavy water (0.5 g). To prevent swamping by the solvent signal and maintain a stable magnetic field strength, the principal corresponding resonances were obtained.

#### 2.3.2 DSC test

5.0–10.0 mg samples placed in standard DSC pans with aluminum lids were tested by PerkinElmer DSC (DSC 6, Norwalk, CT, USA). Samples were heated from room temperature to 200 °C at a rate of 10 °Cmin^-1^ in N_2_ (20 mLmin^-1^). Following that, each scanned sample was cooled to ambient temperature at the same rate, and the thermal changes were recorded.

#### 2.3.3 Microstructure test

Specimens for microstructure testing were prepared by mounting samples (fractured surfaces) of dimensions (0.5 × 0.5 × 0.5 cm^3^) on a metal strap holder, oven dried at 120 °C for 24 h, and then ion sputter-coated with gold (Polaron SC515, Fisons Instruments, UK) at 45 mA for 30 s. Using an ultra-high-resolution field emission scanning electron microscope (UHR-FESEM) (FEI Quanta FEG-650, Netherlands), surface structure parameters were examined at 10 kV, 30°, and 1000× magnification. The percentage elemental compositions of the specimens were determined using the SEM-EDXA (Energy dispersive X-ray spectroscopy analysis) feature of the scanning electron microscope (SEM).

### 2.4 Composite testing and evaluation

#### 2.4.1 Moisture and solid content

An air-dry sample specimen (5 × 5 cm^2^, thickness: 0.5 cm) was weighed into an empty Petri dish and dried in an air-circulating oven at 105 °C for one day. To stabilize the weight, the composite samples were taken out of the oven and cooled inside a desiccator for about 10 min. The weight of each sample was then recorded, and the respective percentage of moisture content was computed.

The solid content of the adhesive was tested with an oven-drying technique. 3.0 g of each sample was weighed in an aluminum pan and dried in an oven at 105 °C for 24 h until no weight changes were observed. After drying, the weight of each sample was recorded, and the average percentage of solid content was determined.

#### 2.4.2 Internal bonding strength

A tensile testing machine (UTM-5582, INSTRON Corporation, Canton, MA, England) with a crosshead speed of 2 mm/min and load cell capacity of $1.0\times 10^{6}$ N was used to measure the internal bonding strength according to ASTM D1037 [24] and JIS A-5908 [25] standard for composites. The cut specimens (5 × 5 cm^2^) were placed between the mould blocks with uniformly spread glued adhesive. The grip specimens were cooled at room temperature and equilibrated at 65% RH for one day. Following that, the loading fixtures were connected to the test machine, and tension was applied vertically to the composite sample surface until the composite failed. For each sample, the system provides a curve that indicates the maximum load at fracture time. The internal bonding strength was computed by taking the ratio of the maximum force required to cause failure per unit area in the bonded composite.

#### 2.4.3 Flexural strength and flexural modulus

Each composite sample was sawed into rectangular pieces (30 × 5 × 1.0 cm^3^) and measurements were taken according to the standard three-point bending test technique at a span-to-depth ratio of 52:1 with a crosshead speed of 10 mm/min using a UTM-5582 testing machine (INSTRON Corporation, Canton, MA, England) based on standard techniques of ASTM D1037 [24] and JIS A-5908 [25]. The test pieces were placed on two supporting points and set at a length of 15 cm, after which the probe was brought into contact with the composite and the preload steadily increased at a constant rate until the sample broke.

#### 2.4.4 Water absorption and thickness swelling

Sample specimens of dimensions 5 × 5 × 0.5 cm^3^ were immersed in distilled water surface at 3 cm and 20 °C at 55% RH for 24 h. After soaking, an electronic balance and digital caliper were used to calculate weight gain and thickness, with the percentage change in weight gain reflecting water absorption and thickness swelling.

#### 2.4.5 Average density using gravimetric and computed tomography (CT) techniques

Gravimetric technique: The composite samples were cut into dimensions (5 × 5 × 0.5 cm^3^) and weighed using a digital weight scale (Metter ToledoTM balance). The dimensional geometry (length, width, and thickness) was ascertained with the aid of a digital caliper (CD-6″ C, Mitutoyo Co., Japan). For each sample formulation, a total of five replicate specimens were tested.

Computed tomography (CT) technique: All samples were scanned using a 16-slice medical X-ray CT scanner (Toshiba Medical System, Canada) with an X-ray peak tube voltage of 120 kVp and 250 mAs. The Adaptive Iterative Dose Reduction 3D (AIDR 3D) algorithm was used during the scanning procedure to minimize radiation dose and noise while optimizing image quality. Using the ImageJ.exe software, the obtained images were analyzed, and a circular region-of-interest (ROI) with a diameter of 2 cm was drawn. Moreover, the calibration relations with reference to tissue substitute plug phantoms (e.g. lung (inhale), lung (exhale), adipose, breast (50/50), water, muscle, and liver) for the CT numbers and density and relative electron density (RED) was achieved using CT electron density phantom CIRS 062 M (Model 062; Computerized Imaging Reference System Inc. CIRS, Norfolk, VA, USA), and the expression is given by Eq. (1) and Eq. (2), respectively.

(1) $$\rho_{120\;kVp}=1.04\times 10^{-3}{CT}_{120\;kVp}+1.02$$

(2) $${RED}_{120\;kVp}=3.5\times 10^{-3}{CT}_{120\;kVp}+3.40$$

where, ${CT}_{120\;kVp}$, $\rho_{120\;kVp}$, and ${RED}_{120\;kVp}$ denote the CT numbers, density, and RED for X-ray peak tube voltage of 120 kVp. The Chi-square ($\chi^{2}$) goodness of fit test display in Eq. (3) was used to determine the difference between each scanned sample from the calculated value of water.

(3) $$\chi^{2}=\sum_{i=1}^{N}{\left(\frac{{CT}_{w_{i}}-{f(CT}_{i})}{\sigma_{i}}\right)}^{2}$$

where, σ is the error in the sample plug phantoms, CT and ${CT}_{w}$ are the CT number of composite and water and i is a positive integer.

### 2.5 Effects of hot-press temperature and time on composite parameters

The specimens were subjected to heat treatment at 155–195 °C for 12–23 min at a moisture content ranging from 6 to 9% using a hydraulic press controlled to within ±1 °C under atmospheric pressure. At the end of each treatment period, the samples were conditioned in a conditioning room at 20 °C and 65% RH and assessed for selected physicomechanical and dimensional stability parameters.

## 3 Results and discussion

### 3.1 Viscosity and pH analysis

**Fig. 1:**
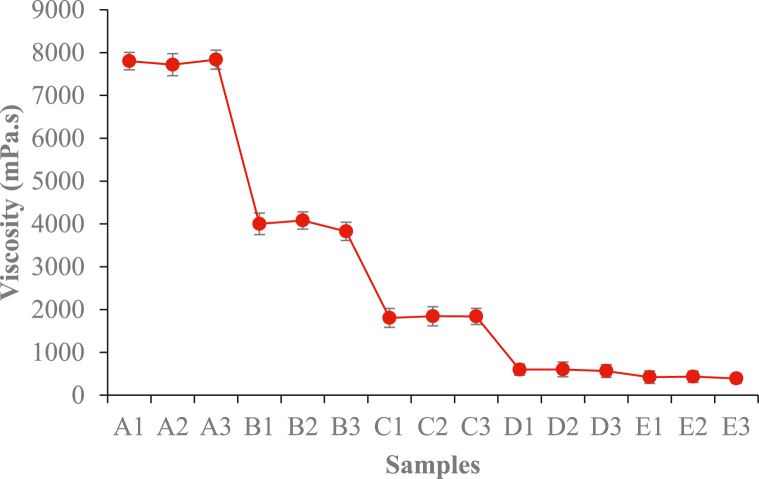
Apparent viscosity at different substitution rates of IA-PAE. Note: A1, A2, A3, B1, B2, B3, C1, C2, C3, D1, D2, D3, E1, E2, and E3 refer to the samples described in Table 1.

The apparent viscosity of the DSF-, SPC-, and SPI-NaOH/IA-PAE adhesives were ascertained for different substitution rates of IA-PAE at room temperature as indicated in Fig. 1. With the increase of IA-PAE substitution rate from 0 wt%–20 wt%, the apparent viscosities of all the adhesives tended to reduce in the range from 7835.70 ± 222.14 mPa s – 391.11 ± 105.82 mPa s, attributed to a decrease in the resistance of the flow of cured DSF, SPC, and SPI suspension. This may also be due to the consequence of the breaking of van der Waals forces between the DSF, SPC, and SPI chains as a result of applied deformation. Samples A1, A2, and A3 are so viscous that they were unable to sufficiently penetrate and provide uneven distribution on *Rhizophora* spp. surface because of the intermolecular interactions, such as electrostatic interaction and disulfide shear between protein molecules. This can result in poor fluidity and inadequate bonding stability. Moreover, significant decreases in the order of: E1/E2/E3 < D1/D2/D3 < C1/C2/C3 < B1/B2/B3 were observed because IA-PAE has a low molecular weight and is in full contact with DSF, SPC, and SPI molecules, which reduces friction between DSF, SPC, and SPI molecular chains. Similarly, average pH values observed during 24.89 °C–27.61 °C ranged from 8.03 ± 0.52–11.11 ± 0.70, classifying the samples as strong alkali substances. This could hasten dissolution and hydrolysis, resulting in the development of high bonding strength in the composites, particularly because pH 11.0 is the best condition for cross-linking reactions [27]. This finding followed a similar trend to that recorded by Gui et al. [16], Fan et al. [22], and Spraul et al. [28], and was closer to the C-PAE-SF, which indicated the presence of self-cross-linking reactions [14,26]. Importantly, as compared to industrial adhesives such as phenol-formaldehyde, these findings are of high quality, suggesting that D1/D2/D3 and E1/E2/E3 are superior in the development of biomaterial adhesives due to their flowability and spreadability.

### 3.2 Physiochemical characteristics

#### 3.2.1 ^1^H NMR analysis

**Fig. 2:**
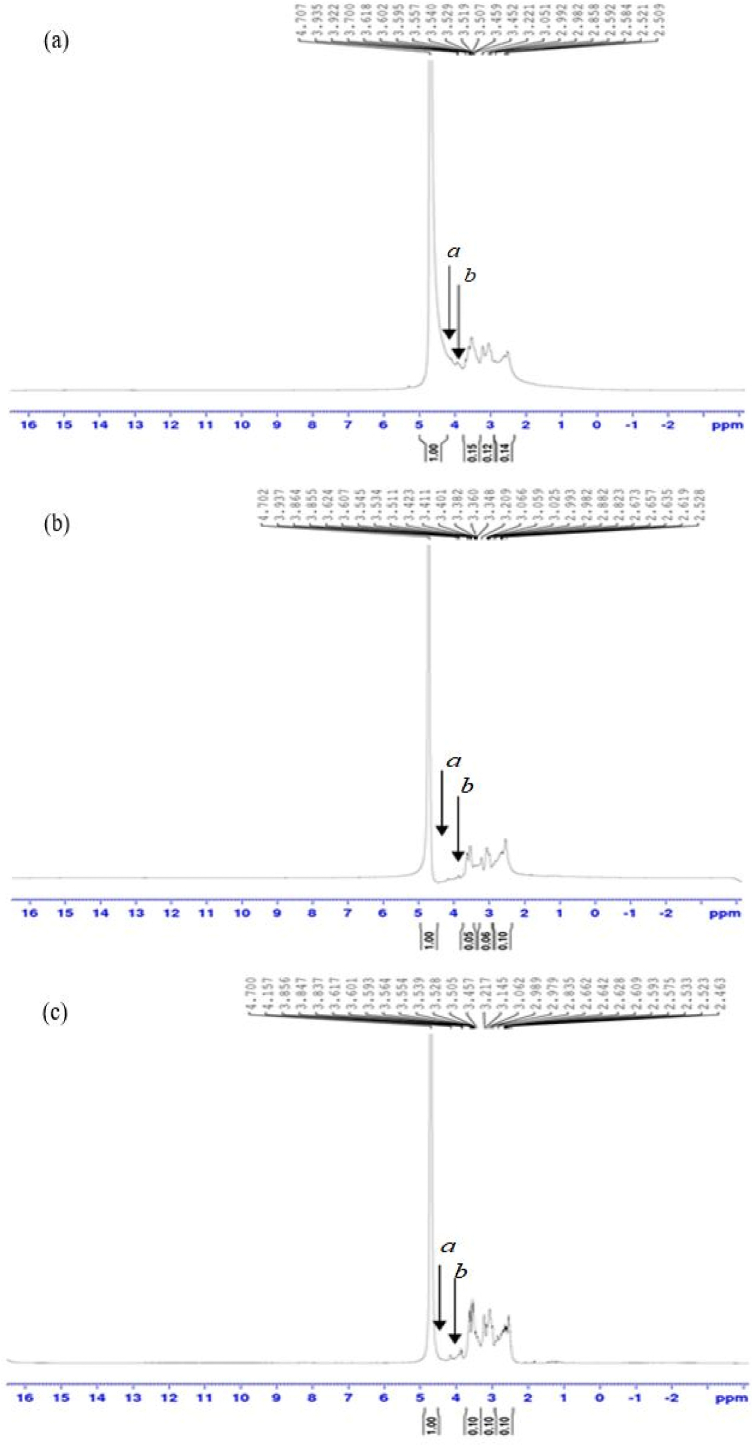
^1^H NMR spectra: (a) DSF/IA-PAE, (b) SPC/IA-PAE, and (c) SPI/IA-PAE.

Fig. 2 shows some typical spectra of the studied adhesive samples. The adhesives are indistinguishable with a broad peak centered at 2.45 ppm–2.80 ppm, assigned to *ortho* and *para* carbon sites. It can be seen clearly that the methine proton *a* of the azetidinium rings functional groups occurred at 4.49 ppm, 4.51 ppm, and 4.54 ppm, with corresponding signals of 3.94 ppm, 3.96 ppm, and 3.95 ppm for DSF/IA-PAE (Fig. 2a), SPC/IA-PAE (Fig. 2b), and SPI/IA-PAE (Fig. 2c), respectively. This can be ascribed to the methine proton *b* of N-(3-chloro-2-hydroxypropyl) groups [14,16]. The adhesives’ similar curing behavior and cured structure suggest that the proposed formulation was capable of producing azetidinium ring functional groups and N-(3-chloro-2-hydroxypropyl) groups, which appear to be able to form viable cross-linking networks with functional groups in DSF, SPC, and SPI [5]. As a result of this finding, a reasonable formulation for the development of low-cost, non-toxic, and high-quality DSF-, SPC-, and SPI-NaOH/IA-PAE adhesives for bonding *Rhizophora* spp. composites could be produced.

#### 3.2.2 DSC analysis

**Fig. 3:**
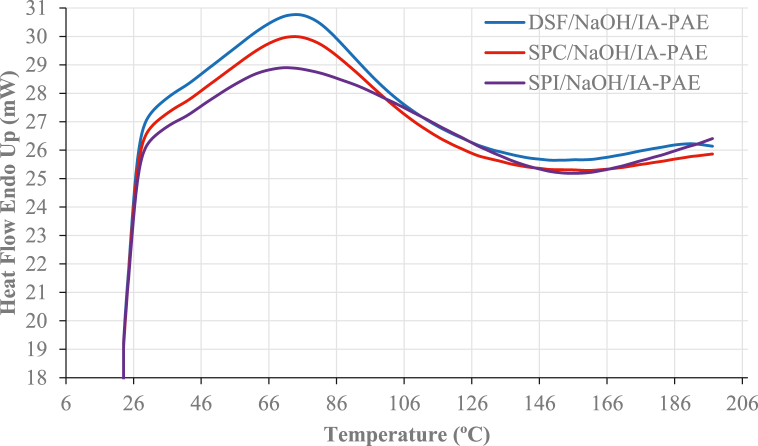
DSC plots.

As shown in Fig. 3, the DSC plots demonstrated denaturation peaks and temperatures imputed to β-conglycinin subunits at 75.10 °C, 74.11 °C, and 71.13 °C, with denaturation enthalpy of 134.66 Jg^-1^, 192.82 Jg^-1^, and 178.76 Jg^-1^, respectively. Denaturation of the DSF, SPC, and SPI by NaOH/IA-PAE exposes the functional groups (OH, COOH, NH_2_, and SH) that can interact with *Rhizophora* spp. particles, resulting in enhanced physical, mechanical, and dimensional stability characteristics [29]. The Figures show two endothermic and one exothermic transition peak corresponding to the conglycinin and glycinin subunits. As can be seen, the endothermic transition peaks were found at 75.10 °C and 190.16 °C for DSF/NaOH/IA-PAE, 74.11 °C and 186.04 °C for SPC/NaOH/IA-PAE, and 71.13 °C and 180.29 °C for SPI/NaOH/IA-PAE. On the other hand, the exothermic peak was found to be 150.17 °C at – 9.289 Jg^-1^, 161.17 °C at – 6.309 Jg^-1^, and 158.52 °C at – 11.534 Jg^-1^, respectively. This finding displayed similarly shaped decomposition plots for both DSF-, SPC-, and SPI-NaOH/IA-PAE/*Rhizophora* spp. because of the chemical cross-linking reaction, which allows thermal energy to be distributed over many bonds, thus, highlighting the formation of a new structure. Summaries of the denaturation peaks and temperatures are shown in Table 2, from DSC measurements.

**Table 2:** Summaries of the denaturation peaks and temperatures.

Samples	Exothermic peaks (°C)	1st Endothermic peaks (°C)	2nd Endothermic peaks (°C)	Denaturation Enthalpy -ΔH (Jg^−1^)	Onset temperature (°C)
DSF/NaOH/IA-PAE	150.17	75.10	190.16	134.66	180.73
SPC/NaOH/IA-PAE	161.17	74.11	186.04	192.82	160.19
SPI/NaOH/IA-PAE	158.52	71.13	180.29	178.76	140.35

#### 3.2.3 UHR-FESEM analysis

**Fig. 4:**
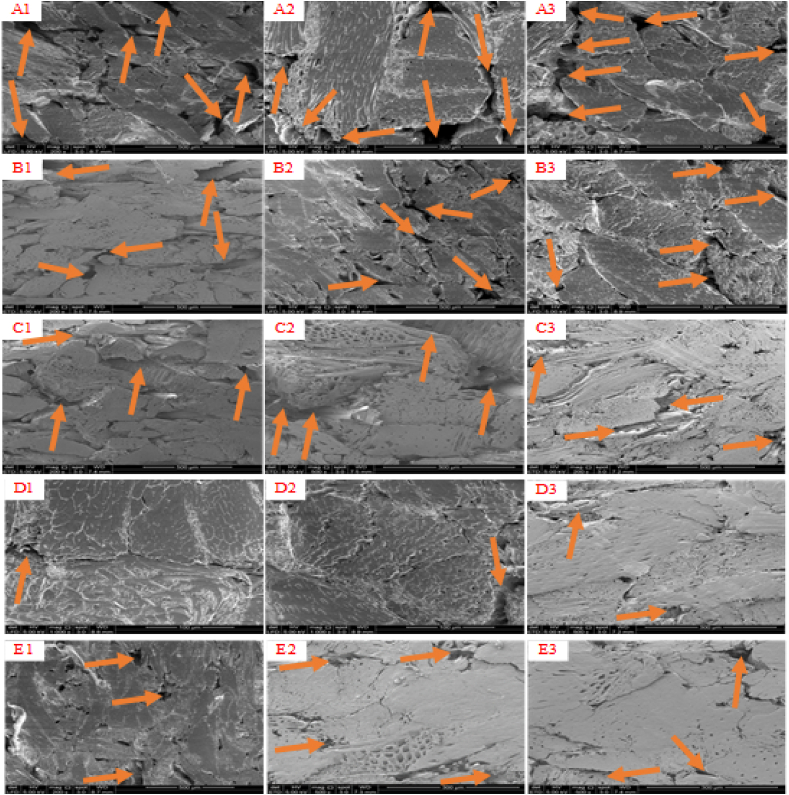
Scanning electron microscopic images of the fracture surface. Note: A1, A2, A3, B1, B2, B3, C1, C2, C3, D1, D2, D3, E1, E2, and E3 refer to the samples described in Table 1.

Fig. 4 demonstrates the microscopic analysis of the composite samples. Many holes and cracks were formed on the fractured surfaces of the composite samples in decreasing order of A1/A2/A3 > B1/B2/B3 > C1/C2/C3 > E1/E2/E3 > D1/D2/D3 through water vaporization of the adhesive during the hot pressing. It can be seen that A1, A2, A3, B1, B2, B3, C1, C2, and C3 showed a cohesive failure, indicating that the adhesives were not homogeneously dispersed. The compression of D1, D2, and D3 results in a homogeneous mixture of cell walls and fibres, implying that the adhesive penetrated deeply into the *Rhizophora* spp. wood, resulting in a smooth surface particle structure on the cross-sections. The void space between the cell lumen and fibres was reduced as a result, suggesting that the NaOH/IA-PAE could efficiently and adequately cross-link the DSF, SPC, and SPI adhesives. These findings are in line with micrographs obtained in previous research [5,8,30]. Consequently, cured DSF-, SPC-, and SPI-NaOH/IA-PAE (15 wt%) biomaterials were the most preferred because of their low viscosity, high pH value, and excellent miscibility, they induced good contact in the *Rhizophora* spp. particles, which contributed significantly to the adhesion of the composite samples. Table 3 has been listed to analyze the chemical composition of A1, A2, A3, B1, B2, B3, C1, C2, C3, D1, D2, D3, E1, E2, E3, water, Perspex, solid water, soft tissue, and paraffin wax. This is important for the construction of a tissue substitute phantom material because the compactness and appropriateness of the material will be ascertained by the cross-linking characteristics of the element present. As can be seen, all of the selected samples had the required elemental parameters relative to those of water and other commercial phantom materials. Since there were no apparent differences in the elements found compared to the composition of human soft tissue, composite samples made of D1, D2, D3, E1, E2, and E3 are ideal and would provide precise feedback on their use as tissue substitute biomaterials.

**Table 3:** Similarity of elemental atomic compositions.

Code	Weight faction of elements (%)													
	H	C	O	N	Na	Mg	P	S	Cl	K	Ca	Mn	Fe	Zn
A1	3.37	50.01	45.57	1.00	–	–	–	0.05	–	–	–	–	–	–
A2	4.02	47.26	48.32	–	–	–	–	0.10	–	–	–	–	–	–
A3	3.88	49.09	47.02	–	–	–	–	0.01	–	–	–	–	–	–
B1	4.52	50.13	44.89	–	0.20	–	0.01	–	–	0.20	0.05	–	–	–
B2	2.85	50.00	46.63	0.27	0.22	–	–	0.03	–	–	–	–	–	–
B3	3.35	49.30	47.01	0.05	0.25	–	–	0.05	–	–	–	–	–	–
C1	4.64	50.01	43.80	1.04	0.04	0.01	0.05	–	0.13	0.05	0.13	–	0.10	–
C2	4.81	48.28	45.00	1.01	0.17	0.07	–	–	0.11	0.20	0.25	0.05	0.05	–
C3	4.93	49.39	43.63	1.09	0.32	0.11	0.09	–	0.15	0.05	0.13	–	0.11	–
D1	5.01	51.94	40.70	1.04	0.21	0.07	0.13	–	0.30	0.19	0.15	–	0.10	0.16
D2	5.36	51.42	39.83	1.60	0.35	0.20	0.20	0.05	0.39	0.20	0.20	–	0.10	0.10
D3	5.29	53.51	39.02	1.11	0.10	0.05	0.21	0.08	0.11	0.14	0.06	0.10	0.14	0.08
E1	4.54	50.02	43.81	1.10	0.09	0.01	0.01	–	0.07	0.20	–	0.04	0.09	0.02
E2	4.78	49.55	44.30	0.92	0.13	0.05	0.01	–	0.05	0.02	0.01	–	0.11	0.07
E3	4.97	50.66	41.91	1.01	0.09	0.11	0.03	0.01	0.10	0.06	0.01	–	0.09	0.05
F^a^	11.11	–	88.89	–	–	–	–	–	–	–	–	–	–	–
G^a^	8.05	59.98	31.97	–	–	–	–	–	–	–	–	–	–	–
H^b^	8.10	67.20	19.90	2.40	–	–	–	–	0.10	–	2.30	–	–	–
I^c^	10.10	11.10	76.20	2.60	0.10	–	0.20	0.30	0.20	0.10	–	–	–	–
J^c^	15.00	85.00	–	–	–	–	–	–	–	–	–	–	–	–

^a^ [31].

^b^ [32].

^c^ [33].

Remark: A1, A2, A3, B1, B2, B3, C1, C2, C3, D1, D2, D3, E1, E2, and E3 refer to the samples described in Table 1, F = water, G = Perspex, H = solid water, I = soft tissue, and J = paraffin wax.

### 3.3 Performance of composite samples

#### 3.3.1 Moisture and solid content analysis

**Fig. 5:**
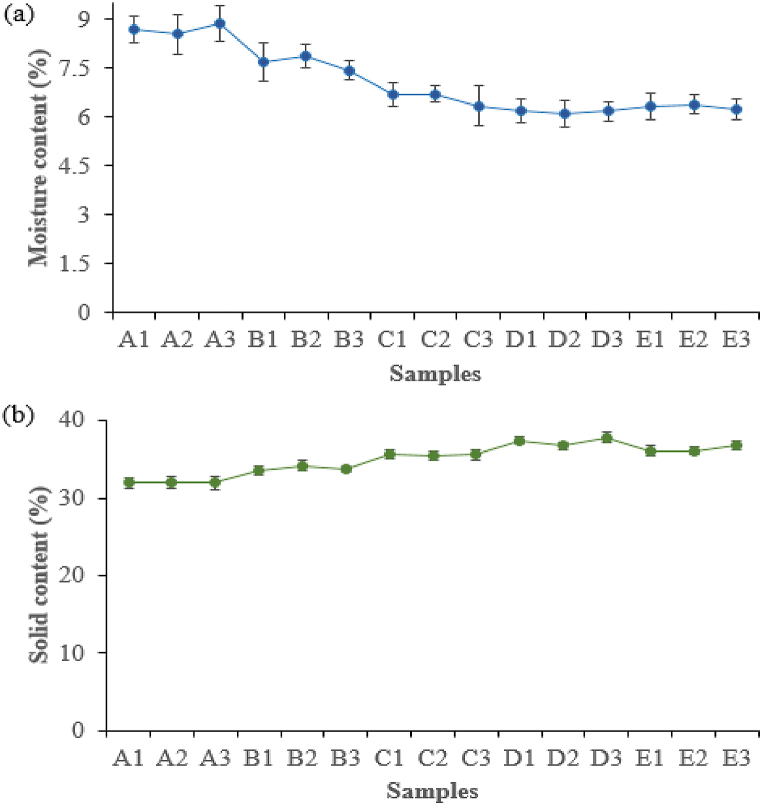
(a) Moisture content and (b) solid content at different IA-PAE substitution rates. Note: A1, A2, A3, B1, B2, B3, C1, C2, C3, D1, D2, D3, E1, E2, and E3 refer to the samples described in Table 1.

*Rhizophora* spp. wood, like many other natural materials, is a hygroscopic substance that absorbs moisture from its surroundings. The amount of absorbed moisture has an effect on various factors that include humidity, type, size, the volume of water, and geometry of *Rhizophora* spp. particles. Overall, the relationship between moisture content and *Rhizophora* spp. has a significant impact on composite sample characteristics and performance. As shown in Fig. 5a, all samples demonstrate good quality values of moisture content that meet the requirements of JIS A-5908 (5–13%) [ 25]. The moisture content percentage levels were found to range from 6.09 ± 0.41%–8.88 ± 0.53%. Comprehensively, the results revealed that improving moisture content followed the order of: D1/D2/D3 < E1/E2/E3 < C1/C2/C3 < B1/B2/B3 < A1/A2/A3, which explains why greater dehydration results in greater material strength in adhesives (D1, D2, D3, E1, E2, and E3). Thus, D1, D2, and D3 give good contact between particles which helps to reduce moisture penetration into the composite. This finding provides further evidence for the proposed adhesion mechanism.

Fig. 5b depicts the solid content of DSF-, SPC-, and SPC/NaOH/*Rhizophora* spp. reinforced with different substitution rates of IA-PAE. As can be shown, adhesives made of NaOH/IA-PAE exhibited significantly higher solid content, making them a promising candidate for adhesive applications based on the reactions of their functional groups. The solid content increased within the range of 31.96 ± 0.80%–37.84 ± 0.65% and achieved its optimum for D1, D2, and D3. In most cases, integrating 0 wt%−5 wt% of IA-PAE into the formulation resulted in a marginally beneficial effect on the solid content, as shown in the Figure. It was discovered that a substitution rate of up to 15 wt% could be achieved without significantly affecting the properties and applicability of the adhesives. As a result, a formulation containing 15 wt% IA-PAE may be considered an adhesive with strong overall properties. These findings are consistent with previous reports of IA-PAE as a cross-linking agent of soy protein-based adhesives [14,16,18,28].

#### 3.3.2 Internal bonding strength analysis

**Fig. 6:**
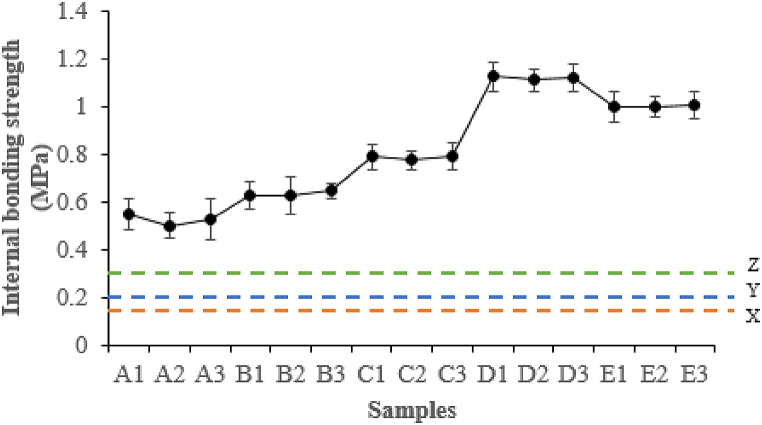
Internal bonding strength of composites at different IA-PAE addition levels. X, Y, and Z represent Type 8, Type 13, and Type 18 of JIS A-5908:2015.

As expected, the internal bonding strength values of the composite samples were observed to increase with increasing substitution rate up to 15 wt% IA-PAE, thereafter decreasing as IA-PAE increased up to 20 wt% (Fig. 6). All particleboards bonded with either DSF- or SPC- or SPI-NaOH/IA-PAE reliably follow the JIS A-5908 and ASTM D1037-99 internal bonding strength requirements for general-purpose particleboards. The highest values were obtained with samples C1, C2, C3, D1, D2, D3, E1, E2, and E3, with D1, D2, and D3 having the optimal quality values easily surpassing types 8, 13, and 18 [25], and superseding what was reported by Zuber et al. [8], Rabaiee et al. [30], and Hamid et al. [34]. This can be ascribed to the observed lesser void spaces, which resulted in increased particleboard strength. This can also be attributed to the more significant presence of high atomic number elements in the composite samples. However, A1, A2, and A3 had the lowest values (0.53 ± 0.09 MPa–0.55 ± 0.01 MPa), owing to their high viscosity, which impedes their application to *Rhizophora* spp. composites. Although some covalent bonding by auto-cross-linking reactions is possible, interfibre bonding is mainly due to hydrogen and other secondary bonds. The improvement of D1, D2, and D3 (1.11 ± 0.05 MPa–1.13 ± 0.06 MPa) is assigned to completely unfurl DSF, SPC, and SPI, which allows the polar functional groups of the side chains to establish interactions with the *Rhizophora* spp. substrates by van der Waals forces, hydrogen bonds, and disulfide bonds. This may also be due to mechanical interlocking caused by reactions in the curing process involving the azetidinium ring functional groups and N-(3-chloro-2-hydroxypropyl) groups.

#### 3.3.3 Flexural strength and flexural modulus analysis

**Fig. 7:**
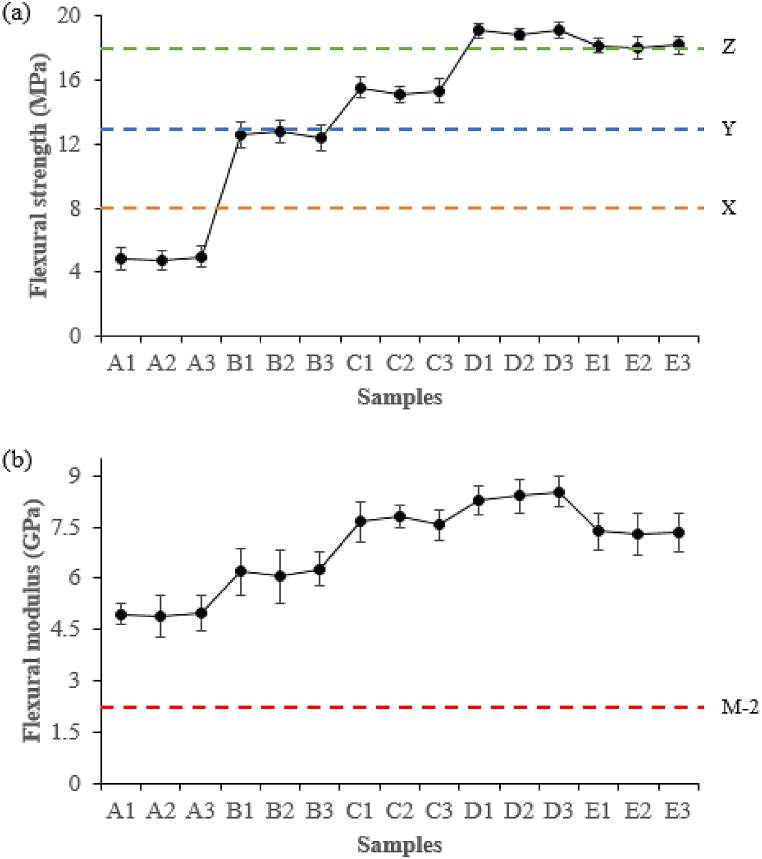
Flexural strength and (b) flexural modulus of composite samples at various content of IA-PAE. X, Y, and Z represent Type 8, Type 13, and Type 18 of JIS A-5908:2015 and M − 2 grade of ASTM D1037-99. Note: A1, A2, A3, B1, B2, B3, C1, C2, C3, D1, D2, D3, E1, E2, and E3 refer to the samples described in Table 1.

Flexural strength and flexural modulus values of different IA-PAE addition levels showed a similar trend as internal bonding strength; the standard minimum flexural strength requirement was easily met for all the investigated samples, as displayed in Fig. 7a. Composite samples with high flexural strength and flexural modulus values are appropriate to accommodate the rigid and heavy workload as phantom materials in radiological areas. The highest flexural strength values were accomplished for samples D1 (19.09 ± 0.44 MPa), D2 (18.87 ± 0.31 MPa), and D3 (19.11 ± 0.50 MPa), which exceeded the JIS A-5908 norm [25] as well as those of previous studies [3,8,30,34]. Moreover, the lowest flexural strength values were found for other samples in the order of: A1/A2/A3 < B1/B2/B3 < C1/C2/C3. It is clear that increasing the IA-PAE to 20 wt% (samples E1, E2, and E3) resulted in a decrease in flexural strength values. This could be attributed to the increased moisture content of the pressed mat composites and higher vapor-gas mixtures at the higher substitution rates of IA-PAE resin. As for flexural modulus, there is no official JIS A-5908 standard for general-purpose particleboards [25], but most of the samples had flexural modulus values between 4.90 ± 0.61 GPa–8.53 ± 0.44 GPa (Fig. 7b) with D1, D2, and D3 having the stronger anti-bending ability, which satisfies the ASTM D1037-99 minimum industrial specifications of 2.25 GPa for M − 2 grade particleboards [24]. Based on these findings, D1, D2, and D3 were the best treatment in terms of flexural strength and flexural modulus, with major increases of 75.14% and 42.56%, respectively, as compared to other selected formulations.

#### 3.3.4 Water absorption and thickness swelling analysis

**Fig. 8:**
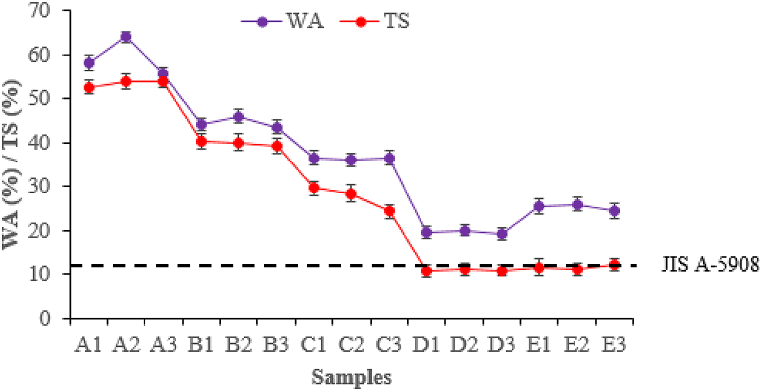
WA and TS effect curve. Note: A1, A2, A3, B1, B2, B3, C1, C2, C3, D1, D2, D3, E1, E2, and E3 refer to the samples described in Table 1.

As presented in Fig. 8, the water absorption (WA) values range from 19.29 ± 1.47%–63.90 ± 1.21%. It is evident that WA decreased gradually as IA-PAE substitution rates increased, eventually stabilizing at 15 wt% (D1, D2, and D3). The lowest values of water absorption were ascertained for D1, D2, and D3 followed closely by E1, E2, E3, C1, C2, and C3, respectively, whereas samples incorporated with 0 wt%–5 wt% (A1, A2, A3, B1, B2, and B3) exhibited the highest values. This may be attributable to the *Rhizophora* spp. hygroscopic nature and its internal structure, which results in a high affinity for WA through capillary action. The lowest WA observed for D1, D2, and D3 can be assigned to the occurrence of polypeptide chains, which reduced the effect of molecular entanglement and exposed more hydrophilic groups. These results are consistent with previous studies [8,12,30]. On the other hand, ideal thickness swelling (TS) values were discovered for 15 wt% and 20 wt% IA-PAE (10.80 ± 1.29%–12.33 ± 1.43%), which met the standard requirements (i.e. below 12%) [25]. However, since samples A1, A2, and A3 (52.57 ± 1.62%–53.95 ± 1.40%) did not minimize the TS of the composites due to the poor fiber moisture-proof ability and large deformation [23], there appears to be no point in using untreated soy proteins alone as a modifier because any hydrophobization of fibres which reduces water absorbance is probably offset by a decline in interfibre and intrafibre bonding that adds to it. This may also be attributed to the observed delamination in the composite samples (A1, A2, and A3) when the hot-press was opened. Therefore, it is clearly seen that D1, D2, D3, E1, E2, and E3 were the most reactive in enhancing the TS of the particleboards because the N-(3-chloro-2-hydroxypropyl) and azetidinium ring groups of IA-PAE reacted with the carboxyl and hydroxyl groups of *Rhizophora* spp. to form a chemical bridge between the DSF-, SPC-, and SPI-*Rhizophora spp*. interface. Overall, the improvement of the WA and TS against moisture could also be attributed to compressing effects of the composite, resulting in the observed high internal bonding strength (D1, D2, and D3).

#### 3.3.5 Average density, CT numbers, and RED analysis

**Fig. 9:**
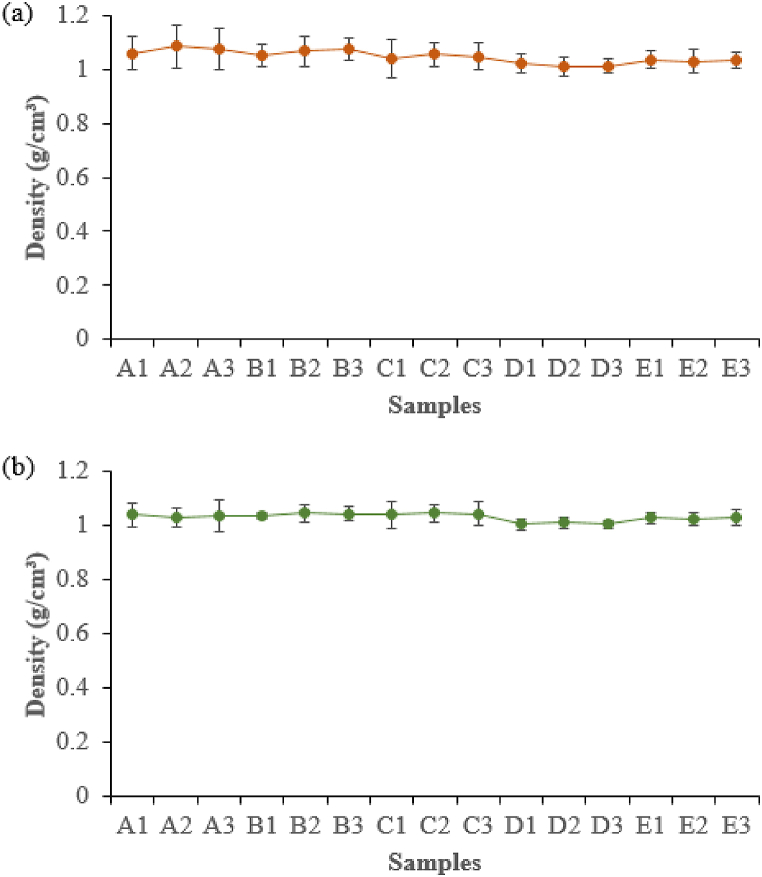
Average density of composite samples using: (a) Gravimetric and (b) CT techniques. Note: A1, A2, A3, B1, B2, B3, C1, C2, C3, D1, D2, D3, E1, E2, and E3 refer to the samples described in Table 1.

The average density values of the composite samples varied based on the IA-PAE substitution rates, which decreased as the moisture content decreased due to water loss during the drying process (Fig. 5a). For both gravimetric and CT techniques, the average density of all selected samples ranged from 1.01 ± 0.04 g/cm^3^–1.09 ± 0.07 g/cm^3^ (Fig. 9a) and 1.00 ± 0.02 g/cm^3^ – 1.04 ± 0.04 g/cm^3^ (Fig. 9b). These results are comparable to those of water (1.00 g/cm^3^), soft tissue (1.03 g/cm^3^), Perspex (1.18 g/cm^3^), as well as of solid water (1.04 g/cm^3^), and are consistent with previous studies [5,8,12,30,33,35]. A comparison of the average densities obtained from the two techniques shows that the CT number was clearly superior to the conventional gravimetric method with a discrepancy of 0.99%–4.59%. The density conversion from the CT number sample method has minimized the uncertainties in the conventional gravimetric technique. This has shown that the CT number provides accurate and reliable estimates of composite density. As can be seen, D1, D2, and D3 have a good average density consistency, which has resulted in the observed close and compact void spaces and can potentially be suggested as an effective tissue substitute material in phantom applications. Therefore, samples D1, D2, D3, E1, E2, and E3 with the appropriate composite performances could be chosen to be tested for the CT numbers and RED characteristics.

The precise correlation relationship between CT numbers and density and their respective electron density of the reference tissue substitute plug phantoms present a good approximation of the electron density accuracy attained through CT imaging. According to Table 4, the equivalent materials (C2, D1, D2, and D3) revealed good and homogenous performance of CT numbers and electron densities values to that of water demonstrated by the calculated total $\chi^{2}$ values (Table 5) compared to A1, A2, A3, B1, B2, B3, C1, C3, E1, E2, and E3. This can be ascribed to their compact uniform surface. The characteristics of D1, D2, and D3 are in good agreement with previous results obtained by Yusof et al. [36], which indicated to be the most effective adhesive formulations to be used in the composites constructed.

**Table 4:** Comparison of mean CT numbers and ED with water.

Samples	Mean CT number	ED × 10^2^³ (electrons/cm³)
A1	−63.89 ± 35.42	3.19 ± 0.33
A2	−75.33 ± 40.59	3.66 ± 0.07
A3	−68.27 ± 35.99	3.47 ± 0.25
B1	−55.71 ± 38.84	3.62 ± 0.71
B2	−43.06 ± 37.22	3.43 ± 0.41
B3	−45.92 ± 35.75	3.30 ± 0.15
C1	−33.49 ± 30.62	3.32 ± 0.26
C2	−29.08 ± 30.75	3.33 ± 0.08
C3	−32.16 ± 30.24	3.32 ± 0.40
D1	−16.94 ± 29.27	3.34 ± 0.21
D2	−21.21 ± 26.50	3.33 ± 0.75
D3	−15.79 ± 28.46	3.34 ± 0.28
E1	−55.52 ± 32.28	3.21 ± 1.11
E2	−44.06 ± 41.03	3.25 ± 0.91
E3	−55.67 ± 35.95	3.21 ± 0.72
Water	−12.29 ± 24.48	3.34 ± 0.20

**Table 5:** $\chi^{2}$ values for CT number to water.

Samples	$\chi^{2}$ values to water
A1	1.751
A2	2.100
A3	1.813
B1	1.025
B2	1.030
B3	1.018
C1	0.243
C2	0.167
C3	0.341
D1	0.029
D2	0.024
D3	0.031
E1	0.175
E2	1.003
E3	0.195

Note: A1, A2, A3, B1, B2, B3, C1, C2, C3, D1, D2, D3, E1, E2, and E3 refer to the samples described in Table 1.

### 3.4 Effects of hot-press temperature and time on physicomechanical and dimensional stability properties

**Fig. 10:**
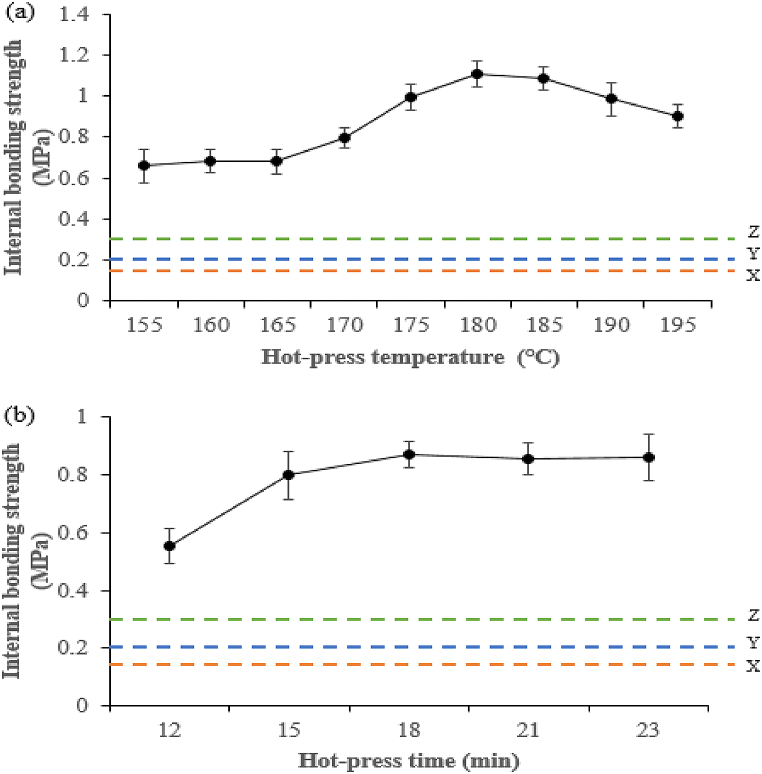
Effect of hot-press on the internal bonding strength of composite samples at different: (a) temperature and (b) time. X, Y, and Z indicate Type 8, Type 13, and Type 18 of JIS A-5908:2015.

The hot-press temperature influences water evaporation, immobilization of DSF, SPC, and SPI molecules, as well as interactions and chemical reactions between DSF-, SPC-, and SPI-NaOH/IA-PAE and the *Rhizophora* spp. surface [37]. As can be clearly seen in Fig. 10, increasing the temperature resulted in increased internal bonding strength. When the hot-press temperature was between 155 and 165 °C, the internal bonding strength remained unchanged, but it increased significantly between 170 and 185 °C, with all samples exceeding the minimum requirements (Fig. 10a). Meanwhile, it was noticed that a further increase in the hot-press temperature up to 195 °C could cause *Rhizophora* spp. and DSF-, SPC-, and SPI-NaOH/IA-PAE adhesives to degrade and have insignificant improvement. On the other hand, the internal bonding strength was lower at a hot-press time of 12 min and significantly increased when the hot-press time ranged from 15 to 23 min because of the high application of heat and pressure during the composite compression (Fig. 10b). In addition, as the hot-press time was increased beyond 21 min, the internal bonding strength was found to be invariant. According to the results, the optimal hot-press temperature and time for completely curing composite bio-adhesives are 180 °C and 18 min. Overall, the hot pressing temperature and time were positively correlated to internal bonding strength.

**Fig. 11:**
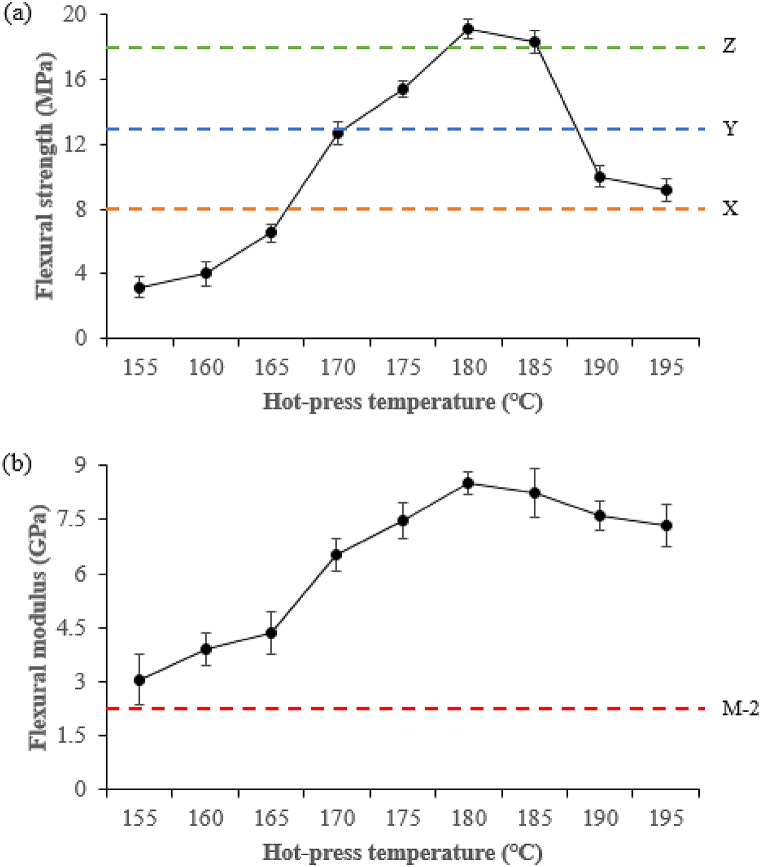
Effect of hot-press temperature on: (a) flexural strength and (b) flexural modulus. X, Y, and Z represent Type 8, Type 13, and Type 18 of JIS A-5908:2015 and M − 2 grade of ASTM D1037-99.

**Fig. 12:**
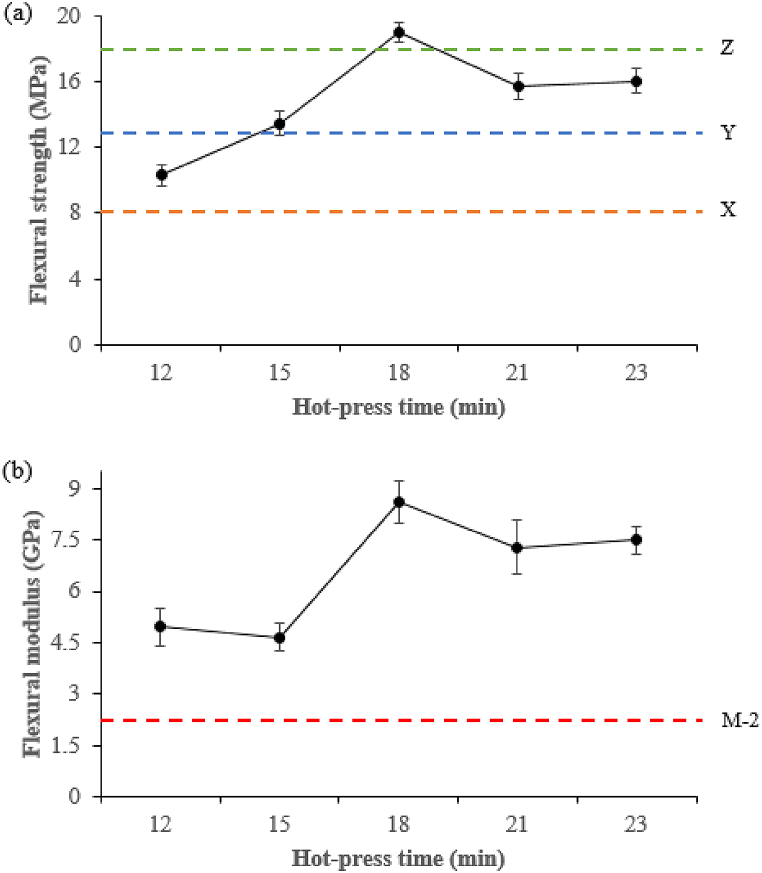
Effect of hot-press time on: (a) flexural strength and (b) flexural modulus.

Fig. 11 highlights the effect of hot-press temperature on flexural strength and flexural modulus. When the temperature was in the range of 155–165 °C, neither flexural strength (Fig. 11a) nor flexural modulus (Fig. 11b) changed significantly, while the optimum values were obtained at 170–185 °C, which exceeded the minimum industrial requirement and resulted in the occurrence of DSF-, SPC-, and SPI-NaOH/IA-PAE adhesives apparent curing reactions, but the values drop sharply from 190 to 195 °C due to the severely burnt and explosion of the composite samples. The effects of hot-press time were presented in Fig. 12. As can be observed, increasing the hot-press time within the range of 12–15 min increased flexural strength (Fig. 12a) and decreased flexural modulus (Fig. 12b), which then increased to their maximum levels when the hot-press time was raised to 18 min, complying with the minimum industrial requirement. Further increasing the hot-press time up to 21–23 min, resulted in composite sample degradation and thus a reduction in strength. Overall, the flexural strength and flexural modulus of the composite samples were improved and appropriate at 180 °C and 18 min.

**Fig. 13:**
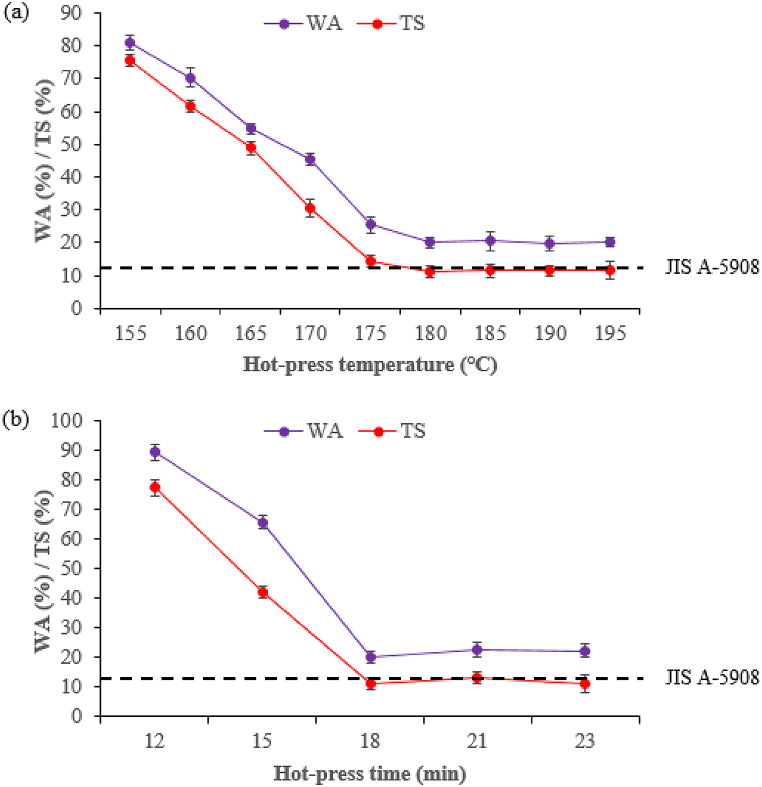
Effect of hot-press on WA and TS of composite samples at different: (a) temperature and (b) time.

The percentage of WA and TS rapidly decreased as the hot-press temperature and time increased from 155 to 170 °C and 12–15 min, then stabilized at 175–195 °C and 18–23 min (Fig. 13a-b). The relatively high WA and TS values are due to the high water intake of the composite samples. In all cases, 180 °C and 18 min gave remarkably better performance.

## 4 Conclusion

The characterization parameters of all the selected samples were found to be satisfactory with comparable structures. The CT number technique was obviously superior to the gravimetric technique, with a difference of 0.99%–4.59%, and the average densities correlated most strongly with those of water, solid water, and human soft tissue. Increasing the substitution rate of IA-PAE above D1, D2, and D3 resulted in a deterioration in moisture content, solid content, internal bonding strength, flexural strength, and flexural modulus, with only a marginal enhancement in water absorption and thickness swelling. This is due to a weak interaction with the protein matrix, as well as the added disadvantages of the composite samples becoming more acidic at E1, E2, and E3. Moreover, composite phantoms (D1, D2, and D3) revealed good and homogenous performance of CT numbers and electron density values to those of water demonstrated by the calculated total $\chi^{2}$ values. Overall, the optimal hot-press temperature and time for completely curing the composite were appropriate at 180 °C and 18 min. We hold the view that the characteristics of D1, D2, and D3 have been demonstrated to be the most effective adhesive formulations to be used in the composites, and that they should be recommended as adhesives for the mangrove wood human tissue substitute materials in medical health applications.

## Author contribution statement

Samson Oluwafemi Damilola, Ph.D.: Conceived and designed the experiments; Performed the experiments; Analyzed and interpreted the data; Contributed reagents, materials, analysis tools or data; Wrote the paper.

Mohd Zahri Abdul Aziz, Ph.D: Conceived and designed the experiments; Analyzed and interpreted the data; Contributed reagents, materials, analysis tools or data.

Ahmad Shukri, Ph.D: Conceived and designed the experiments; Contributed reagents, materials, analysis tools or data.

Nurul Ab. Aziz Hashikin, Ph.D; Abdul Dahiru Addo Buba, Ph.D: Analyzed and interpreted the data.

Rokiah Hashim, Ph.D: Analyzed and interpreted the data; Contributed reagents, materials, analysis tools or data materials-methods.

Mohd Fahmi Mohd Yusof, Ph.D: Performed the experiments; Analyzed and interpreted the data; Contributed reagents, materials, analysis tools or data.

Siti Hajar Zuber, M.Sc.: Conceived and designed the experiments; Performed the experiments.

Sylvester Jande Gemanam, Ph.D; Peter Ayoola Samson, M.Sc: Performed the experiments.

## Funding statement

This research was supported by the Fundamental Grant Research Scheme, Ministry of Higher Education [FRGS/1/2022/STG07/USM/02/2] and Universiti Sains Malaysia Research Grant Scheme [203.PPSK.6171302 & 1001.PPSK.8012381].

## Data availability statement

Data will be made available on request.

## Declaration of interest's statement

The authors declare no competing interests.
